# Effect of testosterone on the Connexin37 of sexual mature mouse cumulus oocyte complex

**DOI:** 10.1186/s13048-016-0290-3

**Published:** 2016-11-23

**Authors:** Yangyang Zhang, Yang Xu, Yanrong Kuai, Sheng Wang, Qing Xue, Jing Shang

**Affiliations:** Department of Obstetrics & Gynecology, Peking University First Hospital, Beijing, 100034 China

**Keywords:** Androgen, Androgen receptor antagonist, Connexin37, Poor ovarian response

## Abstract

**Background:**

Recent researches demonstrate that pre-treatment with androgen could increase retrieved oocytes number and clinical pregnancy rate in poor ovarian response (POR) patients. In view of gap junction intercellular communication (GJIC) is important for follicular growth, and androgen plays an important role in improving prognosis of POR patients, we speculate that androgen can increase the expression of connexin in follicle cells, and improve ovarian microenvironment, thus can promote ovarian response. The objective of the research is to study the effect of testosterone on connexin37 (Cx37) expression so as to provide theoretical basis for adding testosterone in treatment of POR.

**Methods:**

Cumulus-oocyte-cells (COCs) were collected from ICR mice ovaries, and were cultured in vitro for 48 h and then treated with testosterone (T) at various concentration. To assess whether the effect of androgen on Cx37 expression is mediated through androgen receptor (AR) pathway, COCs were cultured in vitro with Flutamide (androgen receptor antagonist). The expression of Cx37 was determined by western blot.

**Results:**

The expression of Cx37 in COCs which were treated with testosterone was higher than that of control group. There were significant differences (*P* < 0.001;<0.001;<0.001;<0.001). Cx37 increased with the elevated testosterone concentrations. Cx37 was lower in androgen receptor antagonist group (2.57 ± 0.12) than the corresponding testosterone concentrations group (4.42 ± 0.28). There were significant differences between two groups (*P* < 0.001).

**Conclusions:**

There was close relationship between gap junction protein and ovarian response, which suggested that androgen could promote ovarian response by increasing the expression of Cx37 in follicle. Androgen plays an important role in ovarian response through the AR pathway and non-AR pathway.

## Background

Poor ovarian response (POR), which is insensitive to ovarian stimulation and results in fewer retrieved oocytes, poorer quality embryos, and lower implantation rate and clinical pregnancy rate, is considered to be one of the most challenging tasks for clinicians in reproductive medicine [1]. POR is common in patients with diminished ovarian reserve (DOR), while the latter is closely related to age. Androgen concentration in ovary decreases sharply as age advances, so we speculate that androgen concentration in follicular fluid of POR patients is lower than normal level. In view of the importance of androgen to follicle recruitment, growth and development, domestic and foreign reproductive centers try to add androgen as an adjuvant to in-vitro fertilization (IVF) treatment protocols in POR patients, in order to increase both quantity and quality of oocytes and embryos, and improve pregnancy outcomes. These interventions include: (1) pretreatment with transdermal testosterone; (2) pretreatment with dehydroepiandrosterone (DHEA); (3) addition of aromatase inhibitors; (4) addition of recombinant luteinizing hormone (LH); (5) addition of human chorionic gonadotrophin (hCG) during ovarian stimulation [2]. Commonly used androgen preparations include transdermal testosterone or oral DHEA. The mechanism of action of androgen in improving ovarian response is unclear. Recent researches report that there is a direct and indirect effect of androgen on follicle development. According to the two cell/two gonadotrophin theory, androgen plays an important role in ensuring adequate follicular steroidogenesis. Produced primarily by the theca cells, it acts as a substrate for the aromatase activity of the granlosa cells, and converts to estrogen (estradiol and estrone), sequentially plays a role in follicle growth [3]. This is called indirect regulation effect of androgen. Recent researches report that androgen can also regulate follicle development directly through various mechanisms [4].

Numerous studies have shown that oocyte is in avascular environment, so normal folliculogenesis relies on the bidirectional talk between any two of granulosa cells, cumulus cells and oocytes [5]. As a form of direct pathway, GJIC plays an important role in folliculogenesis and steroidogenesis [6]. Gap junctions are clusters of intercellular channels composed of two compatible hemichannels (connexin, Cx) in the adjacent cells plasma membrane, which permit the rapid exchange of inorganic ions, second messengers and small molecules from cell to cell. Our previous studies have found that a significant decrease of connexin in cumulus cells is closely related to POR. In view of GJIC is vitally important for follicular recruitment, growth and mature, and androgen plays an important role in improving prognosis of POR patients, so we speculate that androgen can increase the expression of connexin in follicle cells, and improve ovarian microenvironment, thus can promote ovarian response.

Current researches about the effect of androgen on gap junction protein mainly focus on polycystic ovary syndrome (PCOS), but the results are not consistent. Cheng et al. found that high androgen reduces connexin43 expression and impairs GJIC between human granulosa cells. High androgen may impair folliculogenesis and in turn lead to ovulatory dysfunction in PCOS patients [7]. But the result of Rabih et al. was in contrast. The study presented that Cx43 levels were up-regulated in PCO rat ovaries [8]. The researches were aimed at the effect of high concentration of androgen on gap junction protein, and the results still needed further discussion. However, to the best of our knowledge, there has been no report regarding the effect of the different levels with right amount androgen on Cx37 expression in COCs.

The varieties and distributions of connexin in mouse ovary are in accordance with human. Cx43 is mainly localized to the membrane of granulosa cells and cumulus cells, while Cx37 is mainly expressed at the membrane of oocyte in COCs. In this research, we investigated the differences of Cx37 of COCs which were cultured with different concentrations of androgen among physiological level range, and we explored the mechanism of androgen on ovarian response, and provided theoretical basis for adding androgen in treatment of POR in clinical.

The choice of testosterone concentration in culture medium in vitro is mainly on the basis of physiology concentration of androgen in follicle fluid and cytoxicity test in vitro culture of granulosa cells. Researches suggest that the normal concentration of testosterone in follicle fluid is between 10^−8^M and 10^−7^M [9]. Another research about the cytoxicity test in vitro culture of granulosa cells with different concentrations of testosterone suggests that growth inhitibion ratio is about 50% with 10^−4^M testosterone. Therefore, the concentration of testosterone in in vitro culture should be lower than 10^−4^M. We treated COCs with testosterone of the equivalent of physiological concentration in follicular fluid (10^−7^M), low concentration (10^−9^M, 10^−11^M), high concentration (10^−5^M) after comprehensive evaluation from physiology concentration and cytotoxicology test. To assess whether the effect of androgen on connexin was mediated through the AR pathway, we cultured COCs with 10^−7^M testosterone and 10^−6^M Flutamide (androgen receptor antagonist).

## Methods

Twenty six-to-eight-week-old female ICR mice were fed in the barrier environment at animal center of Peking University First Hospital. All animal care requirements were fulfilled, and animals were given food and water ad libitum. After a week, ICR mice were given pregnant mare serum gonadotropin (PMSG, 10 IU per mouse) in order to stimulate ovulation. 48 h later, ICR mice were put to death through dislocating cervical vertebra. The ovaries were removed and washed in sterile conditions. COCs were isolated from the excised ovaries by follicle puncture using needle in culture medium with dissecting microscope. The culture medium was collected and the cells were sedimented by centrifugation at 500 g for 5 min at 4 °C. The COCs were placed in 4-well cell culture plates with 495 μL culture medium at a seeding density of 20–30 COCs per well. COCs were cultured in a humidified incubator (5 % CO_2_) at 37 °C in order to separate from the hormone environment in vivo for 48 h. Then, COCs were treated with 10^−11^mol/L testosterone (T) (dissolved by glycerin, 5 μL), 10^−9^mol/L T, 10^−7^mol/L T, 10^−5^mol/L T, 10^−0^mol/L T (blank control group), 10^−7^mol/L T and 10^−6^mol/L Flutamide for 24 h. The COCs and culture medium were collected respectively and sedimented by centrifugation at 500 g for 5 min. The culture medium was discarded and the COCs were harvested after being washed with cold phosphate-buffered saline (PBS).

Total proteins extracted from COCs were analyzed by western blot for connexin37. Cells were homogenized into RIPA buffer supplemented with protease inhibitors (100:1) for 30 min, and then centrifuged at 3000 g for 20 min. The supernatant was transferred to a new centrifuge tube for western blot. After determination of protein content by the bicinchoninic acid (BCA) protein assay method, 30 μg proteins were separated by 10% sodium dodecyl sulfate polyacrylamide gel electrophoresis (SDS-PAGE) and transferred onto nitrocellulose membranes. After blocked with 5% skimmed milk for 1 h, the membranes were incubated with primary goat anti-mouse Cx37 antibody (1:200, Santa Cruz, USA) for 1 h at room temperature and overnight at 4 °C. After washed three times in Tris-buffered saline (TBS)-0.1% Tween 20, the membranes were incubated with monkey anti-goat IgG conjugated with horseradish peroxidase (HRP) (1:5000, ZSGB-BIO, China) for 1 h at room temperature. The membranes were washed again like above and the immunoreactivity was examined by the enhanced chemiluminescence (ECL) system. As a control for normalization, the membranes were re-probed for internal control, β-actin (1:200, Santa Cruz, USA). Films were scanned, and the optical density of the bands was measured with AlphaEaseFC. The relative quantity of Cx37 was determined with reference to β-actin. Final data were expressed as the mean of results in three independent experiments performed at different time points.

All analyses were performed with Software Package for Social Sciences (SPSS) version 10.0 for windows. All data were expressed as mean ± standard deviation (SD). Comparison between two groups was done with independent sample *T*-test, and comparison among multi samples was done with variance analysis, and intergroup multiple comparison was done with Bonferroni.* P* < 0.05 was considered as statistically significant.

## Results

### Influence of different concentrations of testosterone on Cx37 expression

To study the mechanism of androgen improving ovarian response, our research examined the levels of expression of Cx37 in COCs which were cultured with different levels of testosterone for 24 h in vitro.

**Fig. 1:**
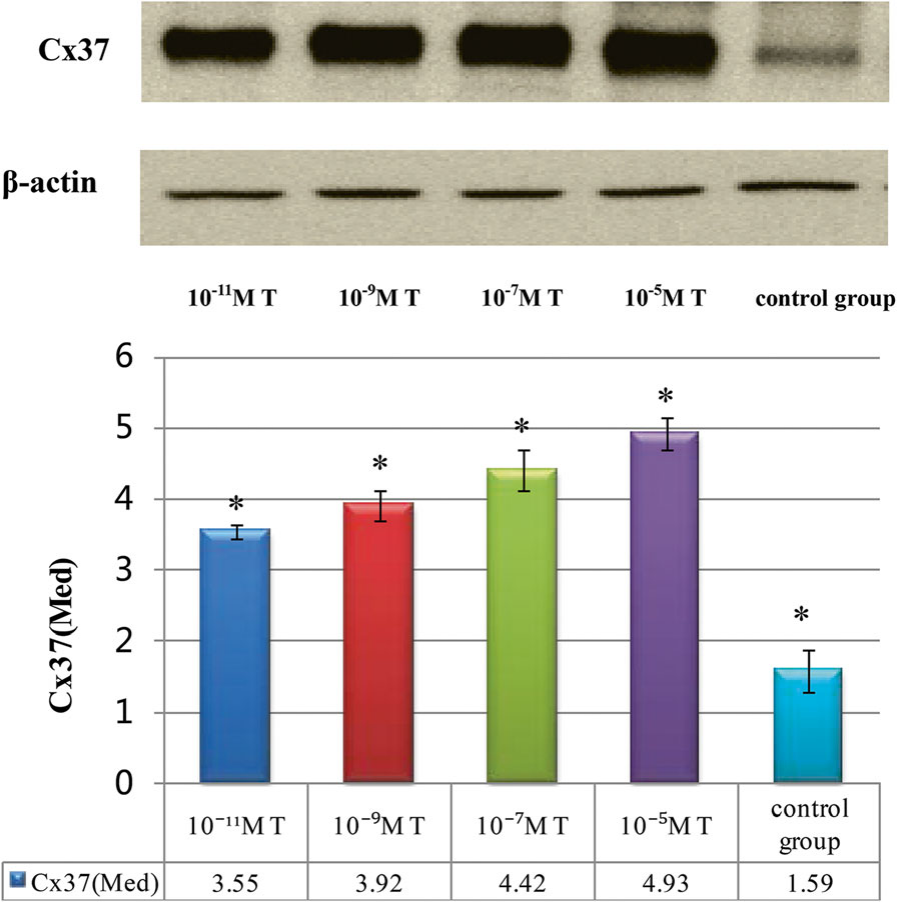
Expression of Cx37 in COCs in different concentrations of testosterone. The expression of Cx37 which were treated with T was higher than that of blank control group. Cx37 increased with the elevated testosterone concentrations, and there was significantly difference in four groups of being cultured with testosterone

Western blot results suggested that Cx37 could be detected in COCs of all groups. The results were shown in Fig. 1. The expression of Cx37 in COCs which were treated with 10^−11^mol/L T (3.55 ± 0.10), 10^−9^mol/L T (3.92 ± 0.21), 10^−7^mol/L T (4.42 ± 0.28), 10^−5^mol/L T (4.93 ± 0.22) was higher than that of blank control group (1.59 ± 0.29). There were significant differences (*P* < 0.001; <0.001; <0.001; <0.001).

The expression of Cx37 in COCs increased with the elevated testosterone concentrations, and there was significantly difference in four groups of being cultured with testosterone (*P* < 0.05). Bonferroni analysis suggested that the expression of Cx37 in COCs which was treated with 10^−11^ mol/L T was significantly lower than those of being treated with 10^−9^mol/L T, 10^−7^mol/L T, 10^−5^mol/L T (*P* = 0.002; <0.001; <0.001). The expression of Cx37 in COCs which was treated with 10^−9^mol/L T was significantly lower than those of being treated with 10^−7^mol/L T, 10^−5^mol/L T (*P* < 0.001; <0.001). The expression of Cx37 in COCs which was treated with 10^−7^mol/L T was significantly lower than that of being treated with 10^−5^mol/L T (*P* < 0.001).

### The effect of androgen receptor antagonist (Flutamide) on Cx37 expression

To assess whether the effect of androgen on Cx37 expression was mediated through androgen receptor (AR) pathway, COCs were cultured in vitro with testosterone and Flutamide. The physiological concentration of testosterone in follicle fluid is 10^−7^M, so we cultured COCs with 10^−7^M testosterone and 10^−6^M Flutamide (10-fold excess above that of testosterone).

**Fig. 2:**
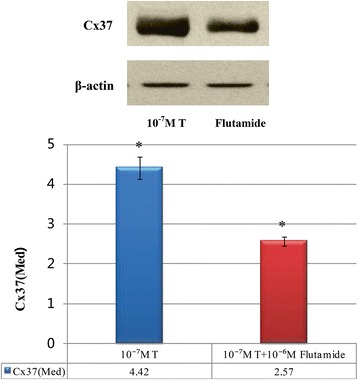
Expression of Cx37 in COCs in androgen receptor antagonist. The expression of Cx37 which were treated with 10^−7^ mol/L T and 10^−6^mol/L Flutamide was lower than that of treated with 10^−7^ mol/L T. There were significant differences between two groups

Western blot results suggested that Cx37 was detected in COCs in both groups. The expression of Cx37 in COCs which were treated with 10^−7^mol/L T and 10^−6^mol/L Flutamide (2.57 ± 0.12) was lower than that of only treated with 10^−7^mol/L T (4.42 ± 0.28). There were significant differences between two groups (*P* < 0.001). The results were shown in Fig. 2.

## Discussion

POR is one of the most challenging tasks for clinicians in reproductive medicine. Several interventions including adjusting stimulation protocols, using oral contraceptive, adding growth hormone have been proposed to improve the POR outcome. Unfortunately, the effect of these interventions is limited. Recent researches with encouraging results demonstrate that pre-treatment with androgen, such as DHEA and trans-dermal testosterone, could increase retrieved oocytes number and clinical pregnancy rate in DOR and POR patients. Casson et al. first reported the benefits of DHEA supplementation for improving the ovarian response and retrieved oocytes number [10]. Since then, a few controlled studies including a randomized controlled study, but with small sample size, have subsequently reported benefits of DHEA supplementation to improve ovarian response and IVF outcome [11]. Nevertheless, controversy still exists as to whether these protocols improved cycle outcome [12, 13]. A recent world-wide survey has shown that over a quarter of reproduction centers add DHEA as an adjuvant to IVF treatment protocols in women with DOR or POR. Despite widespread use of androgen, clinical evidence as well as knowledge regarding underlying mechanisms of androgen on improvement of ovarian response is still limited.

Recent researches report that there is a direct and indirect effect of androgen on follicle development. According to the two cell/two gonadotrophin theory, androgen plays an important role in ensuring adequate follicular steroidogenesis. Produced primarily by the theca cells, it acts as a substrate for the aromatase activity of the granlosa cells, and converts to estrogens (estradiol and estrone), sequentially plays a role in follicle growth [3]. This is called indirect regulation effect of androgen. Recent researches report androgen can also regulate follicle development directly through various mechanisms [4]. The mechanisms include the following several aspects: (1) androgen can increase one or more critical follicle growth factors concentration such as insulin-like growth factor-1 (IGF-1), growth differentiation factor-9 (GDF-9) in follicular fluid during the early stage of follicular development, which are known to have positive effect on follicular development and oocyte quality [14, 15]; (2) androgen can augment granulosa cell follicle stimulating hormone receptor (FSHR) expression and improve follicle sensibility to FSH during the antral follicle stage [16]; (3) androgen via the AR pathway inhibits the expression of PTEN, and phosphorylate Akt proteins by phosphatidyl inositol 3-kinase (PI3K)-Akt signaling pathways, thus induces primordium follicle activation [17]; (4) androgen can up-regulate the expression of AR, promote granulosa cell proliferation and inhibit apoptosis [18].

Our previous studies have found that a significant decreased of connexin in cumulus cells is closely related to POR. In view of gap junctional intercellular communication is vitally important for follicular recruitment, growth and mature, and androgen plays an important role in improving prognosis of POR patients, we speculate that androgen can increase the expression of connexin in follicle cells, and improve ovarian microenvironment, thus promote ovarian response. To test this speculation, we treated COCs with testosterone of the equivalent of physiological concentrations in follicular fluid (10^−7^M), low concentration (10^−9^M, 10^−11^M), high concentration (10^−5^M) and blank control group. The results suggested that compared to control group, testosterone-treated groups demonstrated an increasing amount of Cx37 protein in a dose–dependent manner. Combining with the preliminary study, our research suggested that adding a certain amount of androgen can improve the expression of connexin which is located the surface of oocytes and somatic cells in ovary, strengthen material and information communication among cells, improve ovarian micro-environment, thus improve ovarian response.

However, the mechanism of action of androgen on affecting the expression of connexin is unclear. AR appears on the surface of graunlosa cells when follicles start to improve, and the expression increases with follicle development. The expression of AR increases to peak on the surface of preantral follicle and small antral follicle, then begins to decline, and nearly cannot be detected on the surface of preovulatory follicle [19, 20]. The variation of AR expression suggests that the function of androgen is different at the different stages of follicle development. Androgen can promote follicle recruitment and small antral follicle development through direct action in the early stage of follicle development; while most of androgen translates into estrogen and plays an indirect role in the follicle development, yet there is some direct effect. In this study, the expression of Cx37 in mouse COCs which were treated with androgen receptor antagonist was lower than the corresponding androgen group. The result suggested that androgen via the AR pathway played an important role in Cx37 expression. Further studies are required to understand the androgen-induced GJIC dependent signaling pathways mediating ovarian response.

## Conclusions

This study suggested that androgen can improve the expression of connexin37 which is located the surface of oocytes and somatic cells in ovary in a dose–dependent manner. There was close relationship between gap junction protein and ovarian response, which suggested that androgen could promote ovarian response by increasing the expression of Cx37 in follicle. Androgen via the AR pathway can promote the expression of connexin in COCs, strengthen material and information communication between oocytes and somatic cells, improve ovarian micro-environment, and thus improve ovarian response. The mechanism provides theoretical basis for adding androgen in treatment of POR in clinical. But the problem about rational use of time and dose of androgen still needs large sample clinical research.

## Abbreviations

AR: Androgen receptor
BCA: Bicinchoninic acid
COCs: Cumulus-oocyte-cells
DHEA: Dehydroepiandrosterone
DOR: Diminished ovarian reserve
ECL: Enhanced chemiluminescence
FSHR: Follicle stimulating hormone receptor
GDF-9: Growth differentiation factor-9
GJIC: Gap junction intercellular communication
hCG: Human chorionic gonadotrophin
HRP: Horseradish peroxidase
IGF-1: Insulin-like growth factor-1
IVF: In-vitro fertilization
LH: Luteinizing hormone
PBS: Phosphate-buffered saline
PCOS: Polycystic ovary syndrome
PMSG: Pregnant mare serum gonadotropin
POR: Poor ovarian response
SD: Standard deviation
SDS-PAGE: Sodium dodecyl sulfate polyacrylamide gel electrophoresisSPSS, Software Package for Social Sciences
TBS: Tris-buffered sal
